# Multi-unit relations among neural, self-report, and behavioral correlates of emotion regulation in comorbid depression and obesity

**DOI:** 10.1038/s41598-018-32394-2

**Published:** 2018-09-19

**Authors:** Adam R. Pines, Matthew D. Sacchet, Monica Kullar, Jun Ma, Leanne M. Williams

**Affiliations:** 1 Department of Psychiatry and Behavioral Sciences, Stanford University, Stanford, CA 94305 USA; 2 Department of Medicine, Institute for Health Research and Policy, University of Illinois – Chicago, Chicago, IL 60608 USA; 3 Mental Illness Research Education Clinical, Centers of Excellence (MIRECC), Department of Veterans Affairs (VA), Palo Alto, USA

## Abstract

Depression is a leading cause of disability and is commonly comorbid with obesity. Emotion regulation is impaired in both depression and obesity. In this study, we aimed to explicate multi-unit relations among brain connectivity, behavior, and self-reported trait measures related to emotion regulation in a comorbid depressed and obese sample (*N* = 77). Brain connectivity was quantified as fractional anisotropy (FA) of the uncinate fasciculi, a white matter tract implicated in emotion regulation and in depression. Use of emotion regulation strategies was assessed using the Emotion Regulation Questionnaire (ERQ). We additionally measured reaction times to identifying negative emotions, a behavioral index of depression-related emotion processing biases. We found that greater right uncinate fasciculus FA was related to greater usage of suppression (*r* = 0.27, *p* = 0.022), and to faster reaction times to identifying negative emotions, particularly sadness (*r* = −0.30, *p* = 0.010) and fear (*r* = −0.35, *p* = 0.003). These findings suggest that FA of the right uncinate fasciculus corresponds to maladaptive emotion regulation strategies and emotion processing biases that are relevant to co-occurring depression and obesity. Interventions that consider these multi-unit associations may prove to be useful for subtyping and improving clinical outcomes for comorbid depression and obesity.

## Introduction

Of all the manifestations of depression, one of the most debilitating is that which occurs with comorbid obesity. Depression and obesity are each top contributors to disability and, together, their toll is staggering^1–4^. When they co-occur, depression and obesity are increasingly difficult to treat^5,6^. From a precision medicine perspective^7^, one step toward ultimately developing more targeted interventions is to first understand the brain behavior disruptions that characterize these conditions. Toward this objective, the goal of the current study was to explicate multi-unit relations among neural, behavioral, and trait measures related to emotion regulation, an important component in successful treatment of both conditions^8–10^, in a patient cohort of comorbid depressed-obese individuals.

We focus the current study in diffusion-weighted imaging (DWI) of the uncinate fasciculi, a major white matter tract implicated in emotion regulation, depression, and obesity^11–13^. DWI is a magnetic resonance imaging technique that has been widely used to quantify regional fractional anisotropy (FA), a measure of the directional diffusivity of water; FA is used as a proxy for the structural integrity of white matter tracts^14^. The uncinate fasciculi connect the amygdala and anterior cingulate cortex, as well as other temporal and prefrontal cortical areas^15–17^. In depression, Zhang *et al*.^13^ found significantly lower FA of the right uncinate fasciculus in depressed subjects compared to healthy controls, and Taylor, MacFall, Gerig, & Krishnan^18^ found decreased FA in the left uncinate in early-onset compared to late-onset geriatric depression. DWI studies of obese cohorts suggest a relatively more diffuse reduction of FA in white matter throughout the brain, including the uncinate fasciculi^12,19,20^.

Individual differences in emotional regulation are strong predictors of divergence in treatment outcomes in depression^8,9^ and obesity^10^. These studies have investigated differences in two emotion regulation strategies: expressive suppression (hereafter “suppression”), a type of response modulation in which emotion-expressive behavior is inhibited, and cognitive reappraisal (hereafter “reappraisal”), a type of cognitive modulation in which emotion-related situations are re-constructed to alter emotional impact^21^. The uncinate fasciculi have been related to trait differences in the usage of these strategies. For example, Eden *et al*.^11^ found that higher FA of the left uncinate fasciculus correlated with greater usage of the reappraisal emotion regulation strategy, and Zuurbier *et al*.^22^ found a similar correlation between left uncinate FA and reappraisal in women.

Growing evidence suggests that greater use of suppression (rather than reappraisal) is characteristic of both depression and obesity (e.g.^23,24^). Higher trait usage of suppression has been related to sedentariness and psychopathology (including depressive), while higher usage of reappraisal has been linked to positive outcomes in mental health, weight management practices, relationships, and in the workplace^21,24–30^. Relatedly, depression is associated with abnormal emotional processing, such as heightened attentional capture from negative visual stimuli, better memory for negative material, and difficulty disengaging from negative material^31,32^. Functional neuroimaging studies have linked negative information processing biases in depression to brain areas connected by the uncinate fasciculus, including the amygdala and anterior cingulate cortex^31^, and altered reactivity to negative emotion facial expressions has also been demonstrated to be a predictor of treatment response^33^.

Currently we do not understand how brain structure, usage of specific emotion regulation strategies, and emotion-related behavior relate to one another. Furthermore, our understanding of multi-unit relations among these variables in depression and obesity, separately, is minimal—and data are non-existent in the comorbidity. In the current study, we leveraged data from an ongoing clinical trial^34^ to quantify relations between FA of the uncinate fasciculi, self-reported emotional regulation strategies, and behavioral assays of emotion processing. Our goal was to characterize the relations between these measures in a sample of depressed patients who were obese, with the longer-term view that understanding such relations may help identify “endophenotypes”^35^ and future targets for intervention. Although limited, we drew on prior literature to generate working hypotheses for this study. As previous literature has demonstrated correlations between disadvantageous emotional regulation strategies and FA^13^ we hypothesized that FA of the uncinate fasciculi would relate negatively to suppression and positively to reappraisal strategies in our depressed and obese sample. In this context, as faster reaction times to negatively valenced stimuli have been associated with psychopathology, and reduced FA of the uncinate fasciculi has been related to depression, we further hypothesized that lower FA of the uncinate would be associated with faster reaction times to negative material.

## Results

### Primary sample: ENGAGE Participants

A consort chart detailing data inclusion is included in the supplementary materials (Fig. S1), and participant demographic information is available in Table 1. Of the 108 participants enrolled in the Science of Behavior Change Study (ENGAGE), 77 completed DWI. These patients had moderate to severe depression as assessed by the Patient Health Questionnaire [PHQ-9; 14.10 (*SD* = 3.45)] and an average Body Mass Index (BMI) of 34.92 (*SD* = 4.08), Participants completed their baseline clinical assessment at the Palo Alto Medical Foundation (PAMF), and their baseline imaging visit for DWI at Stanford University. On average, these visits were 50.4 days apart. For those participants who could not complete DWI until their first post-baseline visit, the time since baseline clinical assessment was on average 91.7 days. Of the 77 participants who completed DWI, one did not complete the cognitive battery, and one did not complete the questionnaire battery.

**Table 1:** Demographic information.

	with DWI Acquired	DWI not Acquired	Test Statistic	*p*-value	Effect Size
*N*	77	31			
Race (*χ*^2^)			6.35	0.175	0.243
White	62	19			
Hispanic	7	4			
Black	0	1			
Asian/Pacific Islander/Native Hawaiian	4	4			
Multiple/American Indian/Alaskan Native/Unknown	4	3			
Females/Total (*χ*^2^)	49/77	23/31	0.68	0.408	0.08
Age (*M,SD|t*)	50.8, 11.5	53.3, 12.6	−1.01	0.316	−0.21
BMI (M,SD|t)	34.9, 4.1	37.1, 6.8	−1.66	0.105	0.39
Years of Education (*M|SD|t*)	16.2, 2.8	16.0, 1.9	0.26	0.792	0.05
SCL-20 (Average) (*M|SD|t*)	1.6, 0.6	1.4, 0.5	1.13	0.263	0.24
PHQ-9 (*M|SD|t*)	14.1, 3.5	12.8, 2.1	2.52	0.026^a^	0.45
GAD-7(*M|SD|t*)	8.5, 4.8	6.5, 3.8	2.07	0.041^a^	0.44
ERQ Reappraisal (*M|SD|t*)	26.0, 7.0	25.1, 7.1	0.59	0.559	0.13
ERQ Suppression (*M|SD|t*)	13.9, 5.4	13.0, 3.9	0.88	0.386	0.19
Negative^b^ Reaction Time (*M|SD|t*)	2979.1, 930.2	2907.6, 728.5	0.42	0.675	0.08
Sadness Reaction Time(*M|SD|t*)	2733.3, 1019.8	2472.2, 677.9	1.54	0.128	0.28
Disgust Reaction Time(*M|SD|t*)	2921.3, 1188.6	2777.2, 975.4	0.59	0.556	0.13
Anger Reaction Time(*M|SD|t*)	2945.2, 1070.7	2764.1, 797.9	0.83	0.403	0.18
Fear Reaction Time(*M|SD|t*)	3316.8, 1183.9	3617.0, 1162.9	−1.18	0.239	−0.26
Neutral Reaction Time (*M|SD|t*)	2098.4, 800.0	2907.5, 728.5	−4.81	<0.001^a^	−1.04
Happy Reaction Time (*M|SD|t*)	1612.0, 338.4	1666.4, 412.6	−0.64	0.523	−0.15

DWI = diffusion-weighted imaging; M = mean; SD = standard deviation; *t* = independent samples *t*-test *t*-statistic; *χ*^2^ = *χ*^2^ test statistic; PHQ-9 = Patient Health Questionnaire-9; GAD-7 = Generalized Anxiety Disorder-7; ERQ = Emotion Regulation Questionnaire; ^a^statistically significant; ^b^computed from average reaction time to sadness, disgust, fear, and anger faces. Years of education, PHQ-9 and ERQ Reappraisal scores, and negative, sad, and happy reaction times all had inequivalent variances between groups.

We used the Student’s *t-*test to test for design or clinical characteristics that may contribute to interpretation of the DWI results. If however, an F-test revealed inequality of two variances, we instead used the Welch’s *t*-test; such incidences are noted in-text.

We first assessed whether patients who completed DWI differed in baseline characteristics compared to those who did not. We observed a general equivalence of these two groups, except for higher depression severity (PHQ-9; Welch’s, *t*(84) = 2.52, *p* = 0.014) and anxiety severity (Generalized Anxiety Disorder-7 [GAD-7]; *t*(106) = 2.07, *p* = 0.041) in the DWI completers vs. non-DWI completers. As neither scale correlated with FA of the uncinate fasciculi in the DWI completer group, slightly more severe depression and anxiety was unlikely to account for particular diffusion results in this group. Comparison of DWI patients who completed imaging in the 50.4 versus 90.1 day window revealed significantly higher emotional suppression (Emotional Regulation Questionnaire [ERQ]; *t*(74) = 2.24, *p* = 0.028), and lower depression severity (Symptom Checklist 20 [SCL-20]; *t*(75) = −2.41, *p* = 0.018) in the group who were imaged earlier. Quantification of difference between these groups within our variables of interest are available in Table S3.

### Secondary sample: Healthy comparison Participants

In a secondary comparison sample, we included 19 healthy subjects who were defined as non-clinically depressed according to PHQ-9 scores less than or equal to nine (*M* = 4.38, *SD* = 2.67), and of healthy weight as defined by a BMI of less than 30 (*M* = 21.72, *SD* = 6.16). Consistent with our selection criteria, both the PHQ-9 (*t*(94) = 11.30, *p* < 0.001) and BMI (*t*(94) = 11.27 *p* < 0.001) values were lower in this comparison group than our DWI-imaged depressed and obese group.

### Correlations between FA of the Uncinate Fasciculi and Emotion Regulation Strategies

**Figure 1:**
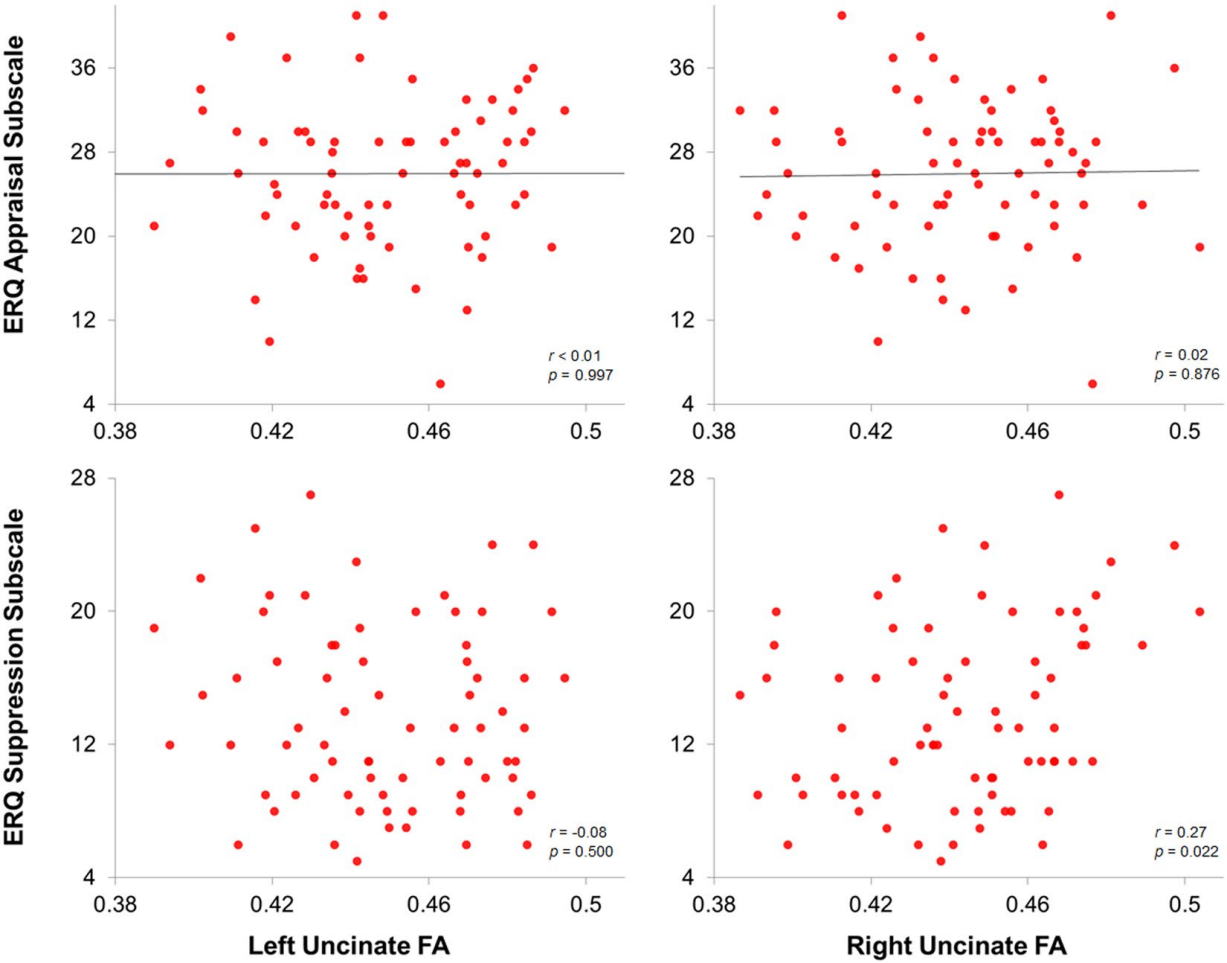
Relations between fractional anisotropy FA of the uncinate fasciculi and trait usage of emotion regulation strategies. Lines of best fit are included for the normally distributed data that was tested using Pearson correlation. Spearman correlation was used for non-normally distributed data. FA of the right uncinate was significantly positively correlated with Emotion Regulation Questionnaire (ERQ) Suppression (*r*_*s*_ = 0.27, *p* = 0.022), but not with reappraisal (*r*_*p*_ = 0.02, *p* = 0.876). FA of the left uncinate was not significant correlated with suppression (*r*_*s*_ = −0.08, *p* = 0.500) or cognitive reappraisal (*r*_*p*_ < 0.01, *p* = 0.997).

All correlations between our variables of interest can be found in Table 2. FA of the right uncinate fasciculus showed a significant positive correlation with Emotion Regulation Questionnaire (ERQ) suppression scores (*r*_*s*_ = 0.27, *p* = 0.022), but not with reappraisal scores (*r*_*p*_ = 0.02, *p* = 0.876). Given group differences in suppression and depression severity in baseline versus follow-up diffusion imaged participants, we sought to examine whether these findings represented participants in both groups. Evaluation of the relationship between suppression and right uncinate FA within time points revealed consistency across baseline (*r*_*s*_ = 0.28, *p* = 0.018) and follow-up visits (*r*_*s*_ = 0.24, *p* = 0.046). FA of the left uncinate fasciculus did not show significant correlation with suppression (*r*_*s*_ = −0.08, *p* = 0.500) or reappraisal scores (*r*_*p*_ < 0.01, *p* = 0.997). See Fig. 1 for scatter plots of these relations. Neither right nor left uncinate FA correlated with scores on the PHQ-9 or GAD-7 (|*r*| ≤ 0.16, *p* ≥ 0.180). Uncinate fasciculi FA and ERQ scores were non-significant in our healthy comparison sample. One observation trended towards significance; ERQ reappraisal and right uncinate FA (*r*_*p*_ = 0.31, *p* = 0.201). Other uncinate-ERQ relations were weak (|*r*_*p*_| ≤ 0.16, *p* ≥ 0.525). Similarly, right and left uncinate FA did not correlate with PHQ-9 scores in this sample (|*r*_*p*_| ≤ 0.22, *p* ≥ 0.357).

**Table 2:** Correlations between variables of interest.

	RU FA	LU FA	BMI	Age	SCL-20	ERQ-R	ERQ-S	Neg RT	Sad RT	Fear RT
RU FA	—	*r*_*p*_ = 0.51 *p* = 2.6 × 10^−6^	*r*_*s*_ = −0.04 *p* = 0.731	r_p_ = −0.19 *p* = 0.102	*r*_*s*_ = 0.03 *p* = 0.813	*r*_*p*_ = 0.02, *p* = 0.876	*r*_*s*_ = 0.27, *p* = 0.022	*r*_*s*_ = −0.26, *p* = 0.026	*r*_*s*_ = −0.30, *p* = 0.010,	*r*_*s*_ = −0.35, *p* = 0.003
LU FA	*r*_*p*_ = 0.51 *p* = 2.6 × 10^−6^	—	*r*_*s*_ < 0.01 *p* = 0.994	*r*_*p*_ = −0.17, *p* = 0.140	*r*_*s*_ = −0.05 *p* = 0.701	*r*_*p*_ < 0.01, *p = *0.997	*r*_*s*_ = −0.08, *p = *0.500	*r*_*s*_ = −0.23, *p* = 0.049	*r*_*s*_ = −0.21 *p* = 0.065	*r*_*s*_ = −0.18 *p* = 0.135
BMI	*r*_*s*_ = −0.04 *p* = 0.731	*r*_*s*_ < 0.01 *p* = 0.994	—	r_*s*_ = −0.33 *p* = 0.003	*r*_*s*_ = 0.14, *p* = 0.221	*r*_*s*_ = −0.17 *p* = 0.152	*r*_*s*_ = 0.03 *p* = 0.824	*r*_*s*_ = −0.23, *p* = 0.045	*r*_*s*_ = −0.14 *p* = 0.210	*r*_*s*_ = −0.32 *p* = 0.005
Age	r_p_ = −0.19 *p* = 0.102	*r*_*p*_ = −0.17, *p* = 0.140	r_*s*_ = −0.33 *p* = 0.003	—	*r*_*s*_ = −0.36 *p* = 0.001	*r*_*p*_ = 0.02 *p* = 0.865	*r*_*s*_ = 0.01 *p* = 0.931	*r*_*s*_ = 0.24, *p* = 0.042	*r*_*s*_ = 0.35, *p* = 0.003	*r*_*s*_ = 0.36, *p* = 0.002
SCL-20	*r*_*s*_ = 0.03 *p* = 0.813	*r*_*s*_ = −0.05 *p* = 0.701	*r*_*s*_ = 0.14, *p* = 0.221	*r*_*s*_ = −0.36 *p* = 0.001	—	*r*_*s*_ = −0.20 *p* = 0.081	*r*_*s*_ = −0.11 *p* = 0.344	*r*_*s*_ = −0.10 *p* = 0.393	*r*_*s*_ = −0.09 *p* = 0.425	*r*_*s*_ = −0.17 *p* = 0.135
ERQ-R	*r*_*p*_ = 0.02, *p* = 0.876	*r*_*p*_ < 0.01, *p* = 0.997	*r*_*s*_ = −0.17 *p* = 0.152	*r*_*p*_ = 0.02 *p* = 0.865	*r*_*s*_ = −0.20 *p* = 0.081	—	*r*_*s*_ = 0.05 *p* = 0.649	*r*_*s*_ = 0.14 *p* = 0.221	*r*_*s*_ = 0.07 *p* = 0.511	*r*_*s*_ = 0.17 *p* = 0.139
ERQ-S	*r*_*s*_ = 0.27, *p = *0.022	*r*_*s*_ = −0.08, *p* = 0.500	*r*_*s*_ = 0.03 *p* = 0.824	*r*_*s*_ = 0.01 *p* = 0.931	*r*_*s*_ = −0.11 *p* = 0.344	*r*_*s*_ = 0.05 *p* = 0.649	—	*r*_*s*_ = −0.11 *p* = 0.362	*r*_*s*_ = −0.06 *p* = 0.612	*r*_*s*_ = −0.11 *p* = 0.337
Neg RT	*r*_*s*_ = −0.26, *p = *0.026	*r*_*s*_ = −0.23, *p* = 0.049	*r*_*s*_ = −0.23, *p* = 0.045	*r*_*s*_ = 0.24, *p* = 0.042	*r*_*s*_ = −0.10 *p* = 0.393	*r*_*s*_ = 0.14 *p* = 0.221	*r*_*s*_ = −0.11 *p* = 0.362	=	*r*_*s*_ = 0.90 *p* = 2.2 × 10^−16^	*r*_*s*_ = 0.87 *p* = 2.2 × 10^−16^
Sad RT	*r*_*s*_ = −0.30, *p* = 0.010,	*r*_*s*_ = −0.21 *p* = 0.065	*r*_*s*_ = −0.14 *p* = 0.210	*r*_*s*_ = 0.35, *p* = 0.003	*r*_*s*_ = −0.09 *p* = 0.425	*r*_*s*_ = 0.07 *p* = 0.511	*r*_*s*_ = −0.06 *p* = 0.612	*r*_*s*_ = 0.90 *p* = 2.2 × 10^−16^	—	*r*_*s*_ = 0.79 *p* = 2.2 × 10^−16^
Fear RT	*r*_*s*_* = *−0.35, *p* = 0.003	*r*_*s*_ = −0.18 *p* = 0.135	*r*_*s*_ = −0.32 *p* = 0.005	*r*_*s*_ = 0.36, *p* = 0.002	*r*_*s*_ = −0.17 *p* = 0.135	*r*_*s*_ = 0.17 *p* = 0.139	*r*_*s*_ = −0.11 *p* = 0.337	*r*_*s*_ = 0.87 *p* = 2.2 × 10^−16^	*r*_*s*_ = 0.79 *p* = 2.2 × 10^−16^	—

r_s_ = Spearmans rho (one or more variables non-normally distributed), r_p_ = Pearson’s correlation RU = right uncinate; LU = left uncinate; FA = fractional anisotropy; BMI = body mass index; SCL-20 = Symptom Checklist 20; ERQ-R = Emotional Regulation Questionnaire Reappraisal subscale; ERQ-S = Emotional Regulation Questionnaire Suppression subscale; Neg RT = aggregate reaction time to negative emotion facial presentation; Sad RT = reaction time to sad emotional facial presentation; Fear RT = reaction time to sad emotional facial presentation.

### Correlations between FA of the Uncinate Fasciculi and Emotion Task Reaction Times

**Figure 2:**
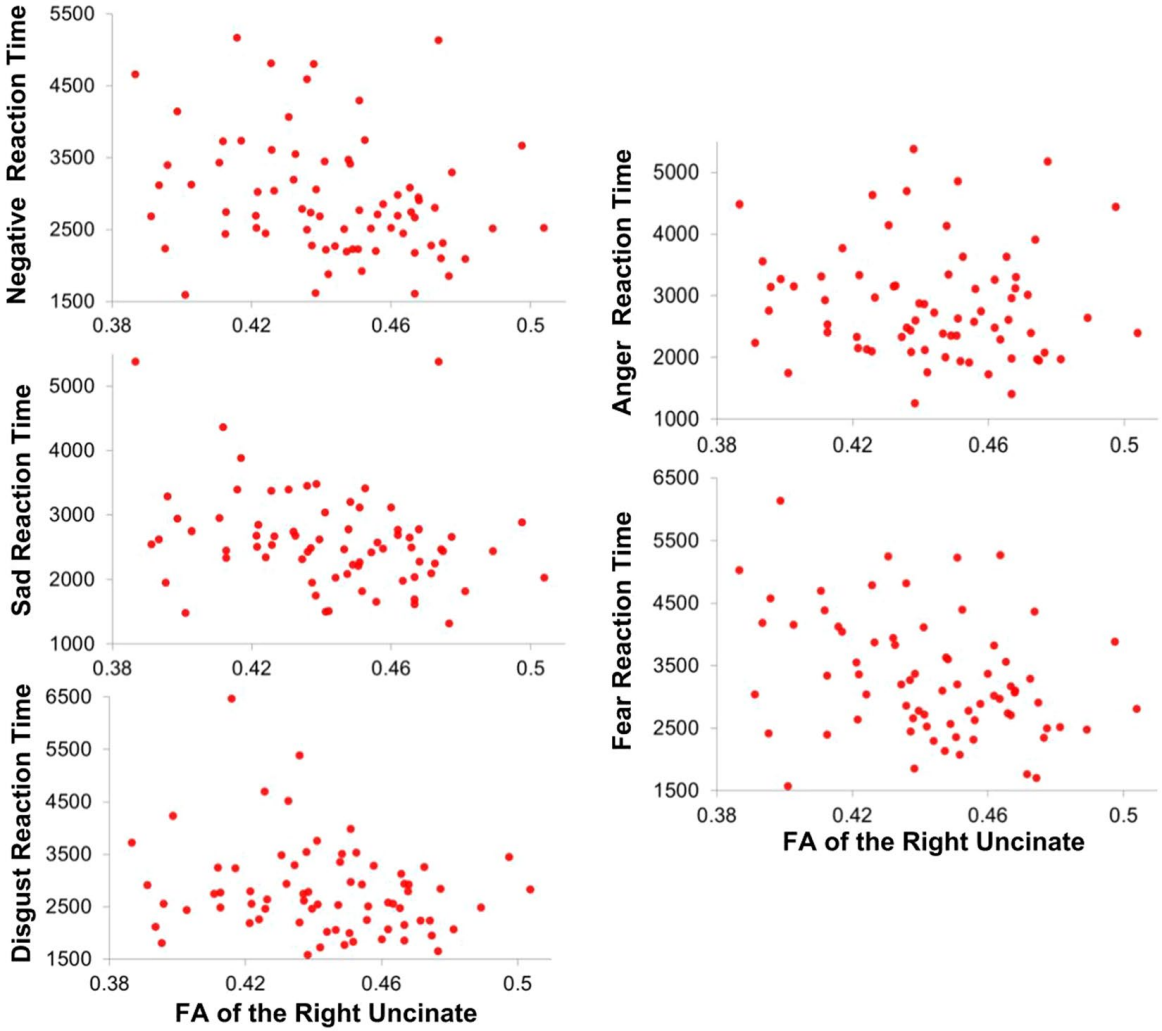
Relations between reaction time to emotional faces (ms) and fractional anisotropy (FA) of the right uncinate fasciculus. As all reaction times were non-normally distributed Spearman correlation was used to assess relations between FA of the uncinate fasciculi and reaction times to negative faces. FA of the right uncinate fasciculus was significantly correlated with reaction time to negative faces (*r*_*s*_ = −0.26, *p* = 0.026). Subsequent analyses revealed that FA of the right uncinate fasciculus significantly negatively correlated with reaction time to sad (*r*_*s*_ = −0.30, *p* = 0.010) and fearful faces (*r*_*s*_ = −0.35, *p* = 0.003). FA of the right uncinate did not significantly correlate with reaction time to disgust faces (*r*_*s*_ = −0.15, *p* = 0.218) or to anger faces (*r*_*s*_ = −0.14, *p* = 0.241).

FA of the right uncinate fasciculus was significantly correlated with reaction time to negative faces (*r*_*s*_ = −0.26, *p* = 0.026), and remained significant after controlling for age (*r*_*s*_ = −0.25, *p* = 0.037). Exploratory analyses revealed that FA of the right uncinate fasciculus was negatively correlated with reaction time to sad faces (*r*_*s*_ = −0.30, *p* = 0.010), and fearful faces (*r*_*s*_ = −0.35, *p* = 0.003) such that higher FA corresponded to faster reaction times. After controlling for age, FA of the right uncinate fasciculus retained a significant negative correlation with sad (*r*_*s*_ = −0.24, *p* = 0.047) and fearful face reaction times (*r*_*s*_ = −0.30, *p* = 0.011). FA of the right uncinate fasciculus did not significantly correlate with reaction time to disgust faces (*r*_*s*_ = −0.15, *p* = 0.218) or to anger faces (*r*_*s*_ = −0.14, *p* = 0.241). See Fig. 2 for scatter plots of these relations. FA of the left uncinate fasciculus did significantly correlate to negative emotion reaction times (*r*_*s*_ = −0.23, *p* = 0.049), although this effect did not remain after controlling for age (*r*_*s*_ = −0.19, *p* = 0.11). Age did not meet our significance threshold when correlated with FA of the right (*r*_*p*_ = −0.19, *p* = 0.102) or left uncinate fasciculus (*r*_*p*_ = −0.17, *p* = 0.140). However, independent of neuroanatomical measures, age did significantly correlate with the composite reaction time to negative emotional faces (*r*_*s*_ = 0.24, *p* = 0.042), sad faces (*r*_*s*_ = 0.35, *p* = 0.003), and fearful faces (*r*_*s*_ = 0.36, *p* = 0.002). Uncinate fasciculi FA and negative reaction times did not demonstrate a relationship within our healthy comparison group (|*r*_*p*_| ≤ 0.16, *p* ≥ 0.51).

### Correlations Between Body Mass Index (BMI) and Variables of Interest

BMI did not relate to FA of the right or left uncinate fasciculi (|*r*_*s*_| ≤ 0.04, *p* ≥ 0.731), severity of anxiety or depression (|*r*_*s*_| ≤ 0.14, *p* ≥ 0.221), nor ERQ subscales (|*r*_*s*_| ≤ 0.17, *p* ≥ 0.152). BMI did significantly negatively correlate with reaction time to identifying negative faces (*r*_*s*_ = −0.23, *p* = 0.045), but this correlation did not survive correction for age (*r*_*s*_ = −0.17, *p* = 0.156). Within our healthy comparison sample, BMI did not relate to uncinate fasciculi FA (|*r*_*p*_| ≤ 0.16, *p* ≥ 0.510) or ERQ scores (|*r*_*p*_| ≤ 0.15, *p* ≥ 0.564).

### Correlations Between Depression Severity and Variables of Interest

Similar to BMI, Symptom Checklist-20 (SCL-20) scores did not significantly correlate with FA of the right or left uncinate fasciculi (|*r*_*s*_| ≤ 0.05, *p* ≥ 0.701), or ERQ suppression scores (*r*_*s*_ = −0.11, *p* = 0.344). Although the relationship between SCL-20 and ERQ reappraisal trended in the expected direction, it did not meet our threshold for statistical significance (*r*_*s*_ = −0.20, *p* = 0.081). SCL-20 scores also did not relate to reaction time to negative faces (*r*_*s*_ = −0.10, *p* = 0.393).

### Control analyses

To assess potential group differences between the treatment and control diffusion-imaged patient groups *within* the ENGAGE project, we conducted two-sample t-tests on group means for our variables of interest. Negative, sadness, and fear reaction were not different at the group level (*t* ≤ 1.17, *p* ≥ 0.248), and neither were ERQ subscale scores (*t* ≤ 0.81, *p* ≥ 0.420), or right and left uncinate FA (*t* ≤ 1.10, *p* ≥ 0.277).

We also conducted two-sample t-tests on the same variables, this time evaluating any potential differences *between* diffusion imaged ENGAGE participants and the healthy comparison sample. Average negative and all individual negative emotion reaction times trended towards slower in the patient group, but none reached statistical significance (t ≤ 1.36, *p* ≥ 0.179). Although patients reported a greater ratio of suppression versus reappraisal (*t* = 3.31, *p* = 0.001), individual ERQ subscale scores did not significantly vary between groups (|*t|* ≤ 1.82, *p* ≥ 0.077). Right and left uncinate FA was similar between groups (*t* ≤ 0.72, *p* ≥ 0.475).

Finally, in order to ensure that software biases and age were not driving our main findings, we removed participants with poor fiber counts and uncinate renderings (*n* = 3). Re-rerunning our analyses with these participants excluded, as well as partial correlations in our parsed sample controlling for age did not change the relationship between right uncinate FA and suppression (*r*_p_ = 0.26, *p* = 0.030). The relations of right uncinate FA to negative, sad, and fear reaction times also remained significant in these conditions (*r* ≤ −0.25, p ≤ 0.037). Full output from these analyses are available in the supplementary materials provided (Tables S1 and S2), as well as a graphical depiction of what constituted a poor uncinate rendering (Fig. S3).

### Regulation Strategy Ratio Analysis

When using the ratio of ERQ suppression score to ERQ reappraisal score, we also found a significant correlation with FA of the right uncinate (*r*_*s*_ = 0.25, *p* = 0.029), including when controlling for age (*r*_*s*_ = 0.28, *p* = 0.018). When performing this analysis on our more stringently quality-controlled sample, we found stronger relationships both before (*r*_*s*_ = 0.29, *p* = 0.015) and after controlling for age (*r*_*s*_ = 0.31, *p* = 0.008). Further detail on the results from this subsample is available in quality-control is available in Tables S1 and S2. We did not find a significant correlation between this ratio and any of our depression and anxiety severity metrics (|*r*_*s*_| ≤ 0.04, *p* ≥ 0.730), reaction time to negative, fearful, or sad faces (|*r*_*s*_| ≤ 0.15, *p* ≥ 0.199), or BMI (*r*_*s*_ = 0.07, *p* = 0.532). The same ERQ subscale ratio did not relate to any variables of interest in our healthy comparison sample, including uncinate fasciculi FA, BMI, PHQ-9 scores, or negative reaction times (|*r*_*p*_| ≤ 0.20, *p* ≥ 0.408).

## Discussion

Structural integrity of an uncinate fasciculus tract that connects brain regions important for emotion regulation was significantly positively correlated with depressed and obese individuals’ tendency to use suppression as an emotion regulation strategy. We observed this relationship in patient population, but not in a non-depressed and healthy weight sample. These findings suggest that in comorbid depressed and obese individuals, greater structural cohesion of the uncinate may be supporting a maladaptive emotion regulation strategy. Our comparison group demonstrated lower suppression relative to reappraisal than our patient sample. This difference suggests less adaptive processing of negative emotion in individuals with concurrent depression and obesity. It is possible that reduced FA in the right uncinate, predictive of both decreased emotional suppression and slower reaction times to negative emotions in our patient sample, represents one neural adaptation by which impedance of emotional information transmission could allow for more time for more advanced emotional processing to occur. More broadly, the coupling between right uncinate FA and deleterious emotional regulation in this patient population suggests altered neural circuitry in comorbid depressed and obese individuals.

Furthermore, we found that the ratio of suppression to reappraisal was a stronger predictor of FA of the right uncinate and captured a greater patient versus comparison difference than suppression scores alone. This indicates that the relative usage of these emotional regulation strategies may be more closely tied to brain structure and pathology than either one independently. These relationships implicate the right uncinate fasciculus in at least two pathways of dysfunctional emotional processes; emotional dysregulation, and increased reactivity to negative emotional stimuli. The presented evidence for a structural phenotype of uncinate FA predicting emotion regulation fits in the context of previous research, where the tract has been implicated in depression and obesity outcomes^13,19,36–40^.

Although unexpected, our findings are intriguing, and justify further inquiry in different and related experimental designs. We speculate that comorbid depressed individuals may have increased function related to the right uncinate fasciculus, leading to top-down suppressive signals sent to medial temporal and orbital brain regions involved in emotion processing. Indeed, several prior studies have reported increased FA in depressed cohorts^38,39^ which may additionally support maladaptive behavior and/or heightened negative emotion. In particular, our study provides evidence of at least one lateralized major white-matter tract as a structural correlate of emotional suppression and negativity bias. Furthermore, because we found no independent relationship of BMI or depression severity to FA in our clinical sample, and did not find the emotional regulation relationships with right uncinate fasciculus FA in our comparison sample, our data demonstrates that these relationships are feasibly not driven by either depression or obesity independently, but rather by the comorbidity of the two. It is worth noting, however, that it is also plausible that when comorbid, the two conditions may interact to alter neural features related to independently occurring conditions, or that this structure-function coupling is only present in groups highly specialized towards suppression as a predominant regulation strategy relative to reappraisal. Both warrant further investigation by comparing different patient populations, particularly independently occurring depression and obesity. Although the cross-sectional nature of our research cannot inform to what extent these correlates may be causal factors of the others, it does establish a basis for a new structural pathway for and within group variation in negative behavioral patterns related to negative emotion perception and regulation.

In our cohort of depressed and obese individuals, we discovered a different relationship between uncinate fasciculi and emotional regulation than had been previously reported in different samples. Previous results demonstrating correlations between FA of the left uncinate and reappraisal^11,22^, and a prior finding that the levels of depressive symptoms positively relate to FA of the right uncinate^13^, were not observed in our study. It should be noted however, that prior studies investigating FA and emotion regulation did not test within an equivalent patient population, and consequently these discrepancies may be attributable to sample heterogeneity. Previous studies have not used BMI and/or depressive symptoms as grounds for inclusion or exclusion; for example, the differences reported by Zhang *et al*.^13^ were between healthy controls and a formally diagnosed major depressive disorder sample, as opposed to our study, which occurred within depressed and obese patients. Although our comparison group did not demonstrate any of these previously-documented relationships between uncinate fasciculi FA and emotional regulation either, we do not propose these non-significant relationships are inarguable evidence against previously obtained findings given the sample size limitation of our comparison group.

Our findings, coupled with that of previous literature, elucidate possible divergence in functions between the left and right uncinate fasciculus. Whereas FA of the left uncinate has been previously positively related to the tendency to use reappraisal as an emotional regulation strategy, as well as decreased anxiety^11,22,41^, our study is the second investigation that has discovered a positive correlation between FA of the right uncinate and maladaptive behavioral expressions in the context of affective disorders^11^. Combined with these previous findings, our study suggests that while increased FA in the left uncinate fasciculus may have psychoprotective effects, increased FA in the right uncinate fasciculus may do the opposite.

We found significant differences in severity of depression and anxiety (as measured by the PHQ-9 and GAD-7) between our diffusion-imaged and non-diffusion-imaged groups. However, as neither scale correlated with FA of the uncinate fasciculus in the DWI group, this group difference in severity of depression and anxiety likely did not influence the observed results. Furthermore, given that the primary focus of this study is emotion regulation, the fact that participants with DWI data had more pronounced depression and anxiety symptoms lessens concerns that null findings may be the result of mild symptomatology. It is also worth noting that the participants who underwent diffusion imaging comprised most our sample, making it difficult to dismiss this group’s relevance to similar clinical populations.

On the other hand, participants who were diffusion imaged at their first visit demonstrated significantly higher baseline suppression and lower baseline depression severity than those who underwent diffusion imaging at their 2-month follow-up. Although this establishes a possibility that these two groups did not have equivalent emotional regulation and uncinate FA relations, we think our relationships of interest being consistent across both samples in spite of reduced sample size is evidence to the contrary.

An additional direction for further research would be to replicate our findings in broader populations, ideally a transdiagnostic sample that includes independently occurring depression and obesity, as well as a larger healthy comparison sample. A more inclusive sample may reveal uncinate structural correlates specific to depression and to obesity, as well as clarifying any interaction effect between depression and obesity on uncinate structural integrity. Future studies could also further probe the relationship between FA of the uncinate fasciculus and harmful emotional processes by investigating whether FA of the uncinate fasciculus longitudinally tracks emotion-related processes relevant to depression and obesity. If this is demonstrated, a subsequent step would be to investigate the utility of the uncinate fasciculus as a biomarker for endophenotyping, more informed patient diagnosis, and/or therapeutic targeting. Additionally, the significant divergence of function we propose between the left and right uncinate fasciculi warrants investigation into their relationship to each other. Because their psychological correlates seem at least somewhat opposed, perhaps the relative dominance of one over the other is a more direct correlate of emotion regulation. If this is the case, a metric such as ratio of right to left uncinate FA may be more indicative of an emotional regulation biomarker than the FA of either tract independently. Finally, investigation into the functional relationships of other major white matter tracts to emotional regulation would elucidate the level of specificity of our findings to the uncinate fasciculi.

Ultimately, our study contributes to understanding neural correlates of depression and obesity. Our findings supplement a host of recent discoveries demonstrating heterogeneity within depressed and obese populations^42,43^, and situate the uncinate fasciculus as a core white matter tract underlying maladaptive emotion regulation strategies in depressed and obese individuals. By furthering our knowledge of biological pathways underlying dysfunctional self-regulatory behavior and providing support of neuroanatomical links that can lead to targeted treatments, we hope to eventually improve treatment outcomes.

## Methods

### Study overview

The current study was conducted within the ENGAGE sample. ENGAGE is an extension of the RAINBOW study using neuroimaging methods to identify potential biomarkers for treatment. The ENGAGE study includes repeated sampling of neuroimaging, virtual reality, behavioral, smartphone, and self-reported questionnaire data. The long-term goal of this study is to contribute to increasing the efficacy of treatments for pervasive and debilitating conditions related to aberrant emotion regulation, including depression and obesity. Further detail on the ENGAGE study is available in a comprehensive protocol paper^34^. Detailed information on the RAINBOW study protocol can be found in previous publications^44^. Briefly, RAINBOW applies a type 1 hybrid design^45^ to evaluate the clinical and cost effectiveness and implementation potential of an integrated, technology-enhanced, collaborative care model for treating comorbid obesity and depression in primary care. The components of the RAINBOW study that are relevant to the current investigation are described in further detail below. The current study includes self-report questionnaire and cognitive-behavioral task data from the ENGAGE baseline visit. Structural neuroimaging (diffusion- and T1-weighted) data was analyzed from either the baseline (*n* = 52) or two-month follow-up visit (*n* = 25), which were six weeks apart. T-tests on baseline vs. two-month follow-up group differences in variables of interest are available in the supplementary materials (Table S3).

### Participant Recruitment

Participants were adult patients (22–76 years old) at PAMF, who had comorbid depression and obesity. All patients were recruited through PAMF primary care clinics. PAMF patients were eligible for RAINBOW and ENGAGE if they were both clinically obese and experiencing current depressive symptoms. All participants were eligible and had been previously randomized to either the experimental or control conditions of the RAINBOW study. Individuals were considered obese if their BMI exceeded 30.0 kg/m^2^ or greater for individuals of non-Asian descent, and 27.0 kg/m^2^ or greater for individuals of Asian descent^44^. Patients were considered depressed if they obtained a score of 10 or greater the PHQ-9^46^. Exclusion criteria are detailed in the supplementary materials, section “ENGAGE Exclusion Criteria”. Written informed consent was obtained from each participant, the Stanford University Institutional Review Board approved the study, and all methods were conducted in accordance with relevant guidelines and regulations.

### Control Analyses

In order to disentangle the potential role that treatment may have played in our findings within variables of interest, we also conducted analyses on group differences between the control and treatment patient groups *within* the ENGAGE study. These participants were randomly assigned to their respective groups in accordance with RAINBOW study protocol^44^.

We also assessed differences between all diffusion-imaged participants within the ENGAGE sample and a healthy comparison sample of 19 participants from a similar neuroimaging study^47^. All healthy comparison participants were scanned at the same facility, with the same exact scanner, and using the identical DWI sequence over the same timeframe as ENGAGE participants. These participants qualified as healthy based on exclusion criteria (PHQ-9 ≤ 9, BMI ≤ 30 for non-Asian descent, BMI ≤ 27 for Asian descent).

In a final control analysis, we removed two participants with right uncinate fiber counts more than three standard deviations below the mean, and the one participant who’s right uncinate fasciculus did not pass manual inspection before rerunning analyses. We also reran these analyses while controlling for age via partial correlations. Full output from these analyses are available in the supplementary materials provided (Tables S1 and S2).

### Depression and Anxiety Severity

The SCL-20 questionnaire is a valid and reliable measure of depression severity^48^. It has been used in numerous trials for depression treatment in primary care and community^49–52^ making it particularly useful for cross-study comparisons and data synthesis in meta-analyses. The SCL-20 was collected at the PAMF during baseline participant visits, approximately two weeks before their fMRI session.

The PHQ-9 has diagnostic validity comparable to clinician-administered diagnosis, and is useful for quantifying the nine criteria that are also used to diagnose major depressive disorder using DSM-IV criteria^44,53^. The PHQ-9 was acquired during the screening of patients for eligibility in the RAINBOW study, and thereby provided an effective proxy for baseline severity of depressive symptoms.

The GAD-7 is a valid and reliable 7-item self-report measure for screening symptoms related to generalized anxiety disorder and has been previously shown to exhibit strong positive relations with multiple domains of functional impairment^54^. The GAD-7 was also collected at the PAMF baseline participant visit.

### Trait Emotion Regulation Strategies

The ERQ is a 10-item self-report questionnaire that has been widely used to assess individual differences in the habitual use of two common emotion regulation strategies: suppression and reappraisal^21^. Participants completed this questionnaire within two days of their ENGAGE neuroimaging visit as a part of a larger self-report battery.

### Behavioral Measures of Emotion Processing

Behavioral measures of emotion processing were obtained from our previously established computerized tests designed to evaluate emotional identification and priming biases by quantifying the speed at which individuals identify facial emotions in images. 48 facial expressions, exhibited from eight different people presenting six emotions (sad, disgust, anger, fear, neutral and happy), were presented twice each on a computer screen^55^. Participants selected (via button press) the perceived emotion, and reaction times were recorded. The emotion identification task was included in the ENGAGE pre-visit battery, similar to the ERQ. Since our hypotheses were specific to negative emotions and to reduce the number of statistical comparisons, for this analysis we considered only negative emotions. Aggregate negative emotion reaction time was calculated by averaging reaction time across the four negative emotions presented in the task (sadness, disgust, anger, and fear). After measuring a significant correlation between aggregate negative emotion reaction time and FA, the aggregate measure and FA were analyzed using partial correlation to account for effects related to age, a known predictor of both variables (e.g.^56,57^). As both variables remained significantly correlated while controlling for age, subsequent exploratory analyses were conducted for individual emotions.

### Body Mass Index

Height and weight were directly measured by trained research staff according to standardized protocol that requires duplicate measurements with no shoes and light clothing at the PAMF baseline participant visit^44^. BMI was calculated as body mass (in kilograms) divided by height squared (in meters).

### Structural Neuroimaging

Whole-brain DWI and T1-weighted images were collected using a 3.0 Tesla GE Discovery MR750 scanner (GE Healthcare, Milwaukee, Wisconsin) with a 32-head channel coil. All scans were conducted at the Stanford Center for Neurobiological Imaging. The T1-weighted images were used for anatomical registration (spoiled gradient echo (SPGR) pulse sequence; repetition time (TR) = 8,656 ms; echo time (TE) = 3.42 ms; flip angle = 11°; resolution = 1.0 mm isotropic; 176 slices; scan duration = 4 min 4 s). DWI was collected using single-shot, dual-spin-echo, echo-planar imaging sequence (84 unique directions; *b* = 1,250 s/mm^2^; TR = 8,700 ms; TE = 8,700 ms; resolution = 2.0 mm isotropic; 70 slices; scan duration = 13 min 29 s). Nine non-diffusion-weighted (*b* = 0 s/mm^2^) volumes were additionally collected for anatomical localization and registration purposes. Because of time constraints, DWI was not collected for every participant in the ENGAGE study.

### Automated Fiber Quantification (AFQ)

AFQ is a recently developed approach to automated DWI tractography clustering^58^. Here we briefly describe the AFQ method for identifying and characterizing major fiber tracts. First, DWI data was preprocessed, including motion correction, data alignment, re-sampling, and trilinear interpolation^59^. Tensors were then fit at each voxel using a robust tensor fitting method^60^, and FA was computed as the normalized standard deviation of the tensor’s eigenvalues. FA ranges from 0 (perfectly isotropic) to 1 (perfectly anisotropic diffusion). Following this, tractography was estimated using a deterministic streamline-tracing algorithm^61,62^. Then, waypoint regions of interest (ROIs) labeled on the Montreal Neurological Institute (MNI) template were warped into participant-specific DWI space, and fibers intersecting these ROIs were identified. A core of each fiber tract was automatically identified. Diffusion metrics were computed along this core, resulting in a “tract profile”. These tract profiles permit the systematic and unbiased assessment of diffusion metrics. In the current study, we analyzed the most commonly quantified diffusion metric: FA. FA for a given tract was computed as the average FA along the tract core.

AFQ identified left and right uncinate fasciculus for 75 of the 77 participants with DWI data. The failure of the software to identify either uncinate on the remaining participants could have been due to noise in the data, crossing fibers, abnormal anatomy, or a limited number of fibers detected within the tracts of interest.

### Data Quality Control

Outliers were removed from reaction time data prior to conducting statistical analyses. Outlier reaction times were defined as scores three or more standard deviations greater or less than the mean. Within the depressed and obese group, we removed one outlier for the combined averaged negative faces reaction times (*z* = 4.05), two outliers for sad faces (*z* = 4.81 and 3.51), two outliers for disgust faces (*z* = 5.02 and 3.31), two outliers for anger faces (*z* = 3.51 and 3.35), and one outlier for fearful faces (*z* = 4.84). Each outlier was removed only for analysis of the variable for which they were more than 3 standard deviations from the mean. The same outlier identification threshold did not yield any outliers within our comparison group reaction times, and no such outliers were observed for ERQ or SCL-20 scores in either group.

Next, we briefly describe several major steps in the AFQ procedure. To reduce noise in the data, AFQ removes fiber lengths that were more than four standard deviations above the mean fiber length, as well as fibers that deviate more than five standard deviations in three-dimensional space from the central portion of their respective core. Additionally, AFQ transforms an established fiber tract probability map^63^ into native subject space. Candidate fibers for tracts that pass through areas of high improbability are discarded. The resulting fibers are only those of physiological feasibility. All uncinate fasciculi were rendered and visually inspected to monitor performance of AFQ tractography clustering. Finally, analyses were conducted after removing data from individuals for which AFQ identified fewer than 300 unilateral uncinate fasciculus fibers (see Supplementary Results: Quality Assurance). This analysis was conducted to assess whether observed effects were influenced by poor clustering performance or low fiber counts.

### Statistical Analysis

Relations among continuous variables (e.g., FA of the uncinate fasciculus, questionnaire scores, reaction times) were assessed using Pearson correlation. Spearman correlation was used when one or more variables were not normally distributed as assessed by the Lilliefor’s test (*p* < 0.05). Correlation coefficients computed using Pearson correlation are indicated with *r*_*p*_, and those computed with Spearman correlation are indicated with *r*_*s*_. Given that our analyses were motivated by *a priori* hypotheses, we did not correct for multiple statistical comparisons.

### Regulation Strategy Ratio Analysis

To test whether observed findings may be driven by individuals with extremes for both evaluated regulation strategies, or rather that observed effects relate to a relative dominance of one strategy over the other, we additionally assessed relations between the ratio of suppression to reappraisal scores and behavioral and neural measures of interest. Outliers on this ratio metric were similarly removed from subsequent analysis. Specifically, three scores were removed (z = 3.29, 3.42, and 4.16) that were three or more standard deviations from the mean across individuals.

## Electronic supplementary material

Supplementary Information
