# Supplementary material for: Multi-unit relations among neural, self-report, and behavioral correlates of emotion regulation in comorbid depression and obesity
Source: Sci Rep. 2018 Sep 19;8:14032. doi: 10.1038/s41598-018-32394-2 (PMC6145883; doi:10.1038/s41598-018-32394-2)

Supplementary Information

Multi-unit relations among neural, self-report, and behavioral correlates of emotion regulation in comorbid depression and obesity

***Adam R. Pines[1], *Matthew D. Sacchet[1], Monica Kullar[1],**

Jun Ma[2], Leanne M. Williams[1,3#]

1. Department of Psychiatry and Behavioral Sciences, Stanford University, Stanford, CA, 94305
2. Department of Medicine, Institute for Health Research and Policy, University of Illinois – Chicago, Chicago, IL, 60608
3. Mental Illness Research Education Clinical, Centers of Excellence (MIRECC), Department of Veterans Affairs (VA) Palo Alto

*contributed equally

Supplementary Methods

RAINBOW-ENGAGE Science of Behavior Change Study (ENGAGE) Exclusion Criteria

Exclusion criteria for the RAINBOW study included active suicidal ideation, any Axis I disorder outside of major or minor depressive disorder as well as comorbidities with any anxiety disorder, active bulimia nervosa within the past three months, active alcohol or substance use disorder, ongoing psychiatric care with a provider outside of Palo Alto Medical Foundation (PAMF), plans to undergo bariatric surgery during the study period or having underwent the surgery in the previous year, pre-existing diabetes (other than during pregnancy), pre-existing cardiovascular disease, diagnosis of cancer (other than non-melanoma skin cancer) that is or was active or treated with radiation or chemotherapy in the past year, diagnosis of a terminal illness and/or residence in a long-term care facility, cognitive impairment (measured from the Callahan 6-item screener[60]), inability to speak, read, or understand English, having no reliable telephone service or no regular Internet access via a computer or mobile device, plans to move out of the study area, current pregnancy or plans to become pregnant during the study period, concurrent enrollment in any other study that could act as a confound, family members of study staff, as well as exclusion at the investigator's discretion for clinical safety or protocol adherence reasons. Exclusion criteria for ENGAGE included a weight limit of 350 lbs, an inability to fit in the MRI system, known neuroanatomical structural abnormalities (e.g., tumor or trauma-induced abnormality), as well as other MRI-related constraints.

Supplementary Results

Quality Assurance

After completion of Automated Fiber Quantification (AFQ), we performed several quality assurance steps to ensure high quality fiber clustering of our data. This included a visual inspection of all rendered uncinate fasciculi. Visual inspection of rendered fiber tracts revealed an irregular right uncinate in one participant. This rendering depicted many fibers that deviated from the core of the fiber tract. We conducted additional analyses removing this individual to ensure that this participant's data did not influence our results.

For quality assurance, we evaluated the calculated number of fibers for all uncinate fasciculi. The two lowest fiber counts were at least 2.07 standard deviations of the mean (*M*=1431.3, *SD*=508.6). To assess their contribution to reported effects we removed these two individuals and conducted additional analyses. After removing the two lowest fiber counts, all correlations we had previously identified remained significant before and after controlling for age (Table S1**)**.

As an additional quality check, we removed one participant from our analysis on the basis of visual inspection of their uncinate rendering (Fig. S3). After adding this step to all previous quality checks (removal of +/-3 standard deviation outliers and bottom 2 fiber counts), all correlations for FA of the right uncinate remained significant before and after controlling for age (Table S2).

Supplementary Tables

Table S1: Correlations between Fractional anisotropy (FA) of the right uncinate fasciculus with behavioral and suppression scores after removing the two lowest fiber count individuals and outliers (|*Z*| > 3). Results are also included for age-corrected partial correlations. All correlations included at least one non-normally distributed variable (as assessed by the Lillifor's Test) and therefore tests were conducted using Spearman correlation.

Neg

N=71	Neg RT	S RT	D RT	A RT	F RT	Suppr
Correlation (r_s *p*)	-0.32	-0.34	-0.18	-0.17	-0.38	0.27
	0.006	*0.004*	*0.143*	*0.172*	*0.001*	*0.022*
Age-corrected (r_s *p*)	-0.27	-0.25	-0.14	-0.13	-0.30	0.28
	0.024	*0.037*	*0.245*	*0.271*	0.*012*	*0.019*

RT=averaged negative reaction time, S RT=sad reaction time, D RT=disgust reaction time, A RT=anger reaction time, F RT=fear reaction time, Suppr=ERQ suppression score.

Table S2: Correlations between Fractional anisotropy (FA) of the right uncinate fasciculus with behavioral and suppression scores after removing the two lowest fiber count individuals, outliers (|*Z*| > 3), and the individual with poorly rendered right uncinate fasciculus. Results are also included for age-corrected partial correlations. Correlations with least one non-normally distributed variable (as assessed by the Lillifor's Test) were conducted using Spearman correlation (indicated by *), otherwise Pearson correlation was used.

Neg

n=70	Neg RT*	S RT*	D RT*	A RT	F RT*	Suppression
Correlation (r *p*)	-0.34<br>*0.004*	-0.35<br>*0.004*	-.19<br>*0.124*	-0.12<br>*0.335*	-0.41<br>*0.001*	0.26<br>*0.030*
Age-corrected (r *p*)	-0.29<br>*0.017*	-.26<br>*0.033*	-0.15<br>*0.212*	-0.09<br>*0.471*	*0.30*<br>*0.012**	0.27<br>*0.028*

RT=averaged negative reaction time, S RT=sad reaction time, D RT=disgust reaction time, A RT=anger reaction time, F RT=fear reaction time, Suppression=ERQ suppression score.

Table S3: Group differences between participants diffusion-imaged at baseline versus 2-month follow up visits. Only sadness reaction time demonstrated inequivalent variances between groups.

	**Baseline Visit (*M*)**	**2-Month follow up (*M*)**	***t***	***p*-value**
N	52	25		
Left Uncinate FA	0.45	0.45	0.12	0.903
Right Uncinate FA	0.44	0.44	0.74	0.465
BMI	35.00	34.75	0.26	0.797
SCL-20 (Average)	1.47	1.78	-2.41	0.018
ERQ Reappraisal	25.92	26.08	-0.09	0.927
ERQ Suppression	14.94	12.08	2.24	0.028
Negative Reaction Time (ms)	2879.61	3168.89	-1.25	0.213
Sadness Reaction Time (ms)	2583.26	2983.14	-1.24	0.224
Fear Reaction Time (ms)	3316.33	3337.34	-0.07	0.944

Supplementary Figures

Figure S1: Consort chart for primary and supplementary analyses. Note that two additional participants were removed due to low fiber counts, and one additional participant was removed due to poor quality fiber clustering of their right uncinate (as assessed by visual inspection). AFQ=Automated Fiber Quantification, RT=Reaction Time, FA=Fractional Anisotropy.

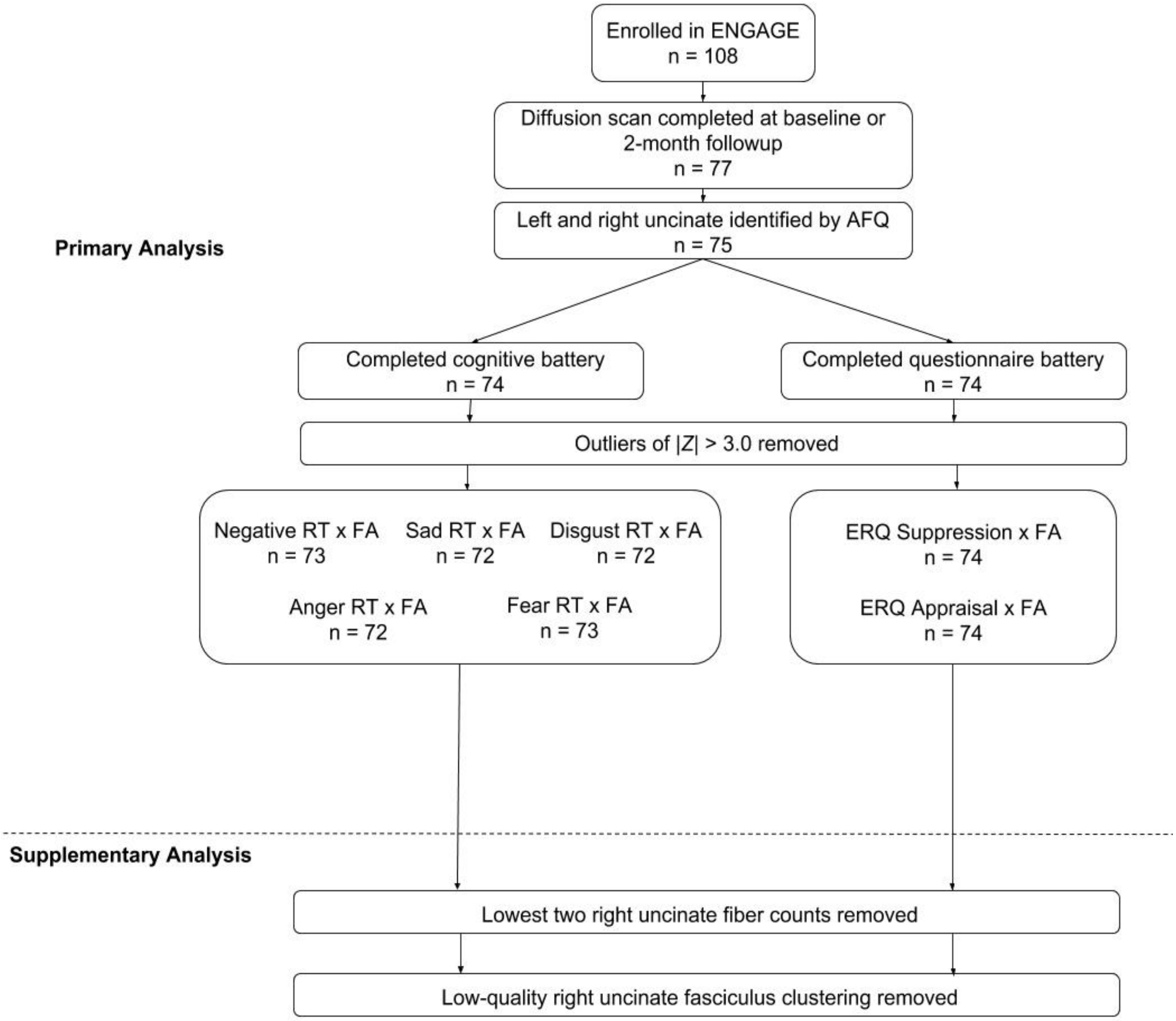

Figure S2: Timeline-format overview of RAINBOW participant enrollment, baseline, and 2-month follow-up ENAGE visits. BV=Baseline Visit, 2MO=2-Month follow-up.

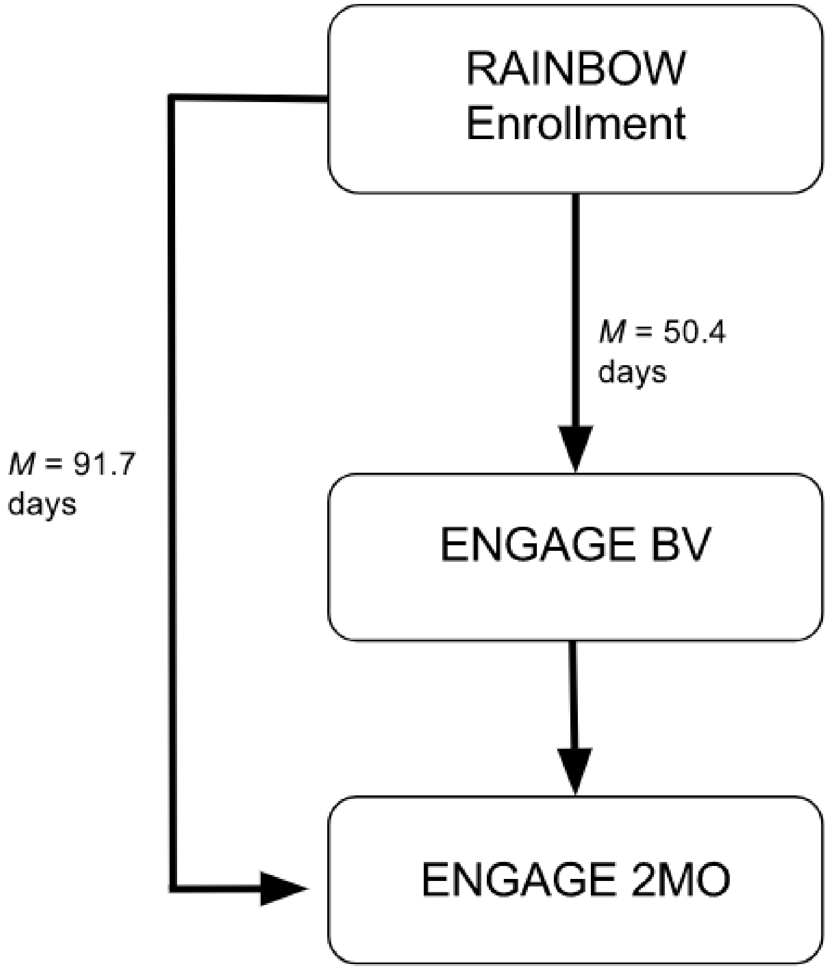

Figure S3: Renderings of high- and low-quality right uncinate fasciculus clusterings. We removed one individuals FA of the right uncinate fasciculus in secondary analyses (left panel). Typical high-quality right uncinate rendering (right panel). Color of tract represents *Z*-score of FA along tract core relative to AFQ reference sample.

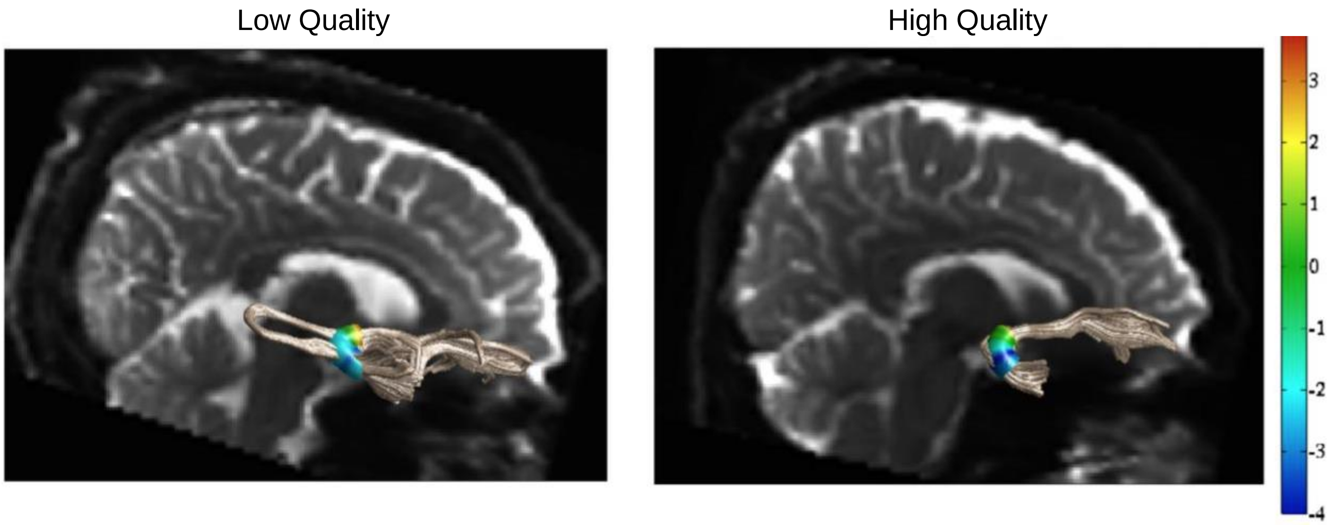

Supplement: Supplementary file 1 — Supplementary Information [file 41598_2018_32394_MOESM1_ESM.pdf]
